# Culturally responsive strategies and practical considerations for live tissue studies in Māori participant cohorts

**DOI:** 10.3389/frma.2024.1468400

**Published:** 2024-11-05

**Authors:** Helena Abolins-Thompson, Kimiora L. Henare, Bridget Simonson, Mark Chaffin, Patrick T. Ellinor, Claire Henry, Mairarangi Haimona, Jake Aitken, Taku Parai, Bianca Elkington, Michael Rongo, Kirsty M. Danielson, Megan P. Leask

**Affiliations:** ^1^Department of Surgery and Anesthesia, University of Otago Wellington, Wellington, New Zealand; ^2^Faculty of Medical and Health Sciences, Molecular Medicine and Pathology, Waipapa Taumata Rau, University of Auckland, Auckland, New Zealand; ^3^Cardiovascular Disease Initiative, The Broad Institute of Massachusetts Institute of Technology (MIT) and Harvard, Cambridge, MA, United States; ^4^Cardiovascular Research Center, Massachusetts General Hospital, Boston, MA, United States; ^5^Department of General Surgery, Wellington Regional Hospital, Wellington, New Zealand; ^6^Te Rōpū Rangahau Hauora a Eru Pōmare, University of Otago Wellington, Wellington, New Zealand; ^7^Te Rūnanga o Toa Rangatira, Porirua, New Zealand; ^8^Department of Physiology, University of Otago, Dunedin, New Zealand

**Keywords:** Indigenous, Māori, ethics, live tissue, genomics, 3D models, snRNASeq

## Abstract

**Introduction:**

Indigenous communities globally are inequitably affected by non-communicable diseases such as cancer and coronary artery disease. Increased focus on personalized medicine approaches for the treatment of these diseases offers opportunities to improve the health of Indigenous people. Conversely, poorly implemented approaches pose increased risk of further exacerbating current inequities in health outcomes for Indigenous peoples. The advancement of modern biology techniques, such as three-dimensional (3D) *in vitro* models and next generation sequencing (NGS) technologies, have enhanced our understanding of disease mechanisms and individualized treatment responses. However, current representation of Indigenous peoples in these datasets is lacking. It is crucial that there is appropriate and ethical representation of Indigenous peoples in generated datasets to ensure these technologies can be used to maximize the benefit of personalized medicine for Indigenous peoples.

**Methods:**

This project discusses the use of 3D tumor organoids and single cell/nucleus RNA sequencing to study cancer treatment responses and explore immune cell roles in coronary artery disease. Using key pillars from currently available Indigenous bioethics frameworks, strategies were developed for the use of Māori participant samples for live tissue and sequencing studies. These were based on extensive collaborations with local Māori community, scientific leaders, clinical experts, and international collaborators from the Broad Institute of MIT and Harvard. Issues surrounding the use of live tissue, genomic data, sending samples overseas and Indigenous data sovereignty were discussed.

**Results:**

This paper illustrates a real-world example of how collaboration with community and the incorporation of Indigenous worldviews can be applied to molecular biology studies in a practical and culturally responsive manner, ensuring fair and equitable representation of Indigenous peoples in modern scientific data.

## Introduction

Globally, Indigenous communities are affected by high incidences of non-communicable diseases. Cancer and cardiovascular disease in particular are amongst the leading causes of mortality and morbidity and are recognized as health priorities by these communities (Garvey and Cunningham, 2019; Segelov and Garvey, 2020; Curtis et al., 2022; Te Aho o Te Kahu, 2023). The driving factors for these health inequities are complex and multi-factorial. Lasting impacts of colonization and institutional racism account for factors such as poor access to clinical care, poor pathways to diagnoses, and mistreatment in the healthcare system (Curtis et al., 2023; Reid and Robson, 2007; Griffiths et al., 2016; Selak et al., 2020). Importantly, improvements are being made to address some of these issues, including; increasing Indigenous participation in screening programs, embedding cultural values into health-related policy and science systems, and building capacity for non-Western models of health (Curtis et al., 2022; Henare et al., 2019; Reid et al., 2017). As treatment strategies advance and change, it is important that the implementation of Indigenous worldviews continues to be addressed. Currently, there is an increasing focus on personalized medicine approaches for the treatment of non-communicable diseases. This offers both an opportunity for improving the health of Indigenous people, and a high risk of further exacerbating existing inequities in health outcomes through poorly developed and implemented approaches (Matheson et al., 2018; Claw et al., 2018, 2024; Tsosie et al., 2021a,b). As advances in molecular biology techniques continue to improve our understanding of disease pathology and personalized treatment approaches, it is crucial that Indigenous participants are represented appropriately in generated datasets to ensure the technologies being used serve Indigenous populations in an equitable manner. By doing so, we can maximize the benefits of personalized medicine and genomic technologies for these communities.

Our scientific understanding of the underlying biological mechanisms of diseases such as cancer and cardiovascular disease has improved dramatically due to the development of complex *in vitro* models of diseases and advances in sequencing technology (Bi et al., 2021; Boretto et al., 2019; Brazovskaja et al., 2019; Kim et al., 2020; Chaffin et al., 2022; Ding et al., 2020; Fan et al., 2021; Simonson et al., 2023). In cancer research, the development of patient-derived 3D tumor organoids enables more accurate representation of the range of cells present in the tumor, the tumor microenvironment, and tumor architecture *in vitro* (Saeki et al., 2023; Porter et al., 2020; Spagnol et al., 2023; Tuveson and Clevers, 2019). Treatment of tumor organoids with cancer therapeutics allows for a greater understanding of therapeutic response compared to more traditionally used 2D culture models (de Witte et al., 2020; Narasimhan et al., 2020; Sisman et al., 2022). Coupled with next generation sequencing (NGS) technologies such as single-cell and single-nucleus RNA sequencing (scRNA-seq/snRNA-seq), we have the ability to understand the underlying transcriptional effect of cancer and cancer treatments at an individual cell level (Tuveson and Clevers, 2019). These technologies are now being used for the discovery of novel drugs and to better tailor therapies to stratified patient groups for more personalized treatment approaches (Fan et al., 2021).

In cardiovascular disease, snRNA-seq technology has allowed work to be carried out on human cardiac tissues to understand the transcriptional shifts that occur in patients with heart disease, and to identify which cell types these shifts occur in Chaffin et al. (2022), Simonson et al. (2023), Reichart et al. (2022), Litvinukova et al. (2020), Tucker et al. (2020), Amrute et al. (2023), and Liu et al. (2022). Large scale whole genome sequencing and GWAS studies have also been used to understand how underlying genetic mutations cause and impact development of heart disease, including; atrial fibrillation (Roselli et al., 2018), dilated cardiomyopathy (Garnier et al., 2021), and coronary artery disease (Mudd-Martin et al., 2021; Kalayinia et al., 2018; Parikh and Ashley, 2017; Aragam et al., 2022). Together, this information allows identification and stratification of patient populations that are at a higher risk for heart disease and development of personalized medicines that target genes in the relevant cell type for treatment.

Currently, most studies using these techniques and technologies severely lack representation of Indigenous participants. This is a well-documented issue for genome wide-association studies (Claw et al., 2018; Mudd-Martin et al., 2021; Need and Goldstein, 2009; Popejoy and Fullerton, 2016; Mills and Rahal, 2019; Martin et al., 2019) and may have accuracy implications when interpreting NGS gene panel tests involving Indigenous peoples in personalized medicine contexts. It also can lead to the development of therapies that are not beneficial for Indigenous populations, and missed opportunities for identifying therapies that would be beneficial for Indigenous populations (Nagaraj and Toombs, 2021; Ortega and Meyers, 2014; Patrinos et al., 2023; Nguyen et al., 2020; Henderson et al., 2019; Samarasinghe et al., 2023; Beyene et al., 2021). Thus, unless this inequity in research is addressed, the application of these technologies in health research and health care will ultimately exacerbate health inequities for Indigenous populations (Tawfik et al., 2023; Gwynne et al., 2023).

To improve Indigenous participation with and in research it is necessary to understand the distinctions in Indigenous knowledge and culture so that research can be conducted in a culturally respectful and safe manner. For example, for Māori (the Indigenous peoples of Aotearoa New Zealand) traditional knowledge of one's whakapapa is crucial to assertions of Māori identity and tribal membership. The cultural practice of song (waiata), proverbs (whakatauki), and ritual chant (karakia) ensures that the knowledge of whakapapa is preserved and passed from generation to generation accurately through oral tradition (Te Rito, 2007; Connor, 2019; Roberts, 2013). Regrettably, traditional Indigenous knowledge has historically been ignored due to the domineering assertion of Western science's written form of knowledge transfer (Stevens, 2008). This lack of acknowledgment of Indigenous traditional knowledge has contributed to mistrust in Western science practices (Aramoana et al., 2020; Beaton et al., 2017). However, understanding the significance of whakapapa (genealogy) for example, allows one to understand the importance and taonga (precious) nature of genetic material. This aids understanding of the previous failures of researchers (Garrison et al., 2019) and the mistrust and reluctance that surrounds biomedical research due to scientific colonialism and misappropriations. Importantly, Indigenous peoples have strong and well-informed perspectives on key functional parts of translational research such as biobanking, tissue banking and genomics-based research (Claw et al., 2018; Beaton et al., 2017; Hudson et al., 2016a; Caron et al., 2020), which demonstrate the importance of an individual feeling culturally safe in their research contributions (Beaton et al., 2017; Hudson et al., 2016a; Caron et al., 2020; Hudson et al., 2016b; Reweti et al., 2023; Hudson et al., 2016c). Cultural safety, in its most simplistic form, is often described as a step beyond cultural competence, whereby systematic steps toward decolonizing and transforming health research is performed in a way that respects the needs, rights and identities of Indigenous peoples (Curtis et al., 2019; Tremblay et al., 2023). As scientists, we need to understand and respect cultural practices in all aspects of research, to ensure research is conducted in a manner that protects and empowers our Indigenous participants that contribute to it.

In Aotearoa, there is the }responsibility to Te Tiriti o Waitangi (The Treaty of Waitangi), of Māori and non-Māori individuals, to work together toward eliminating entrenched health inequities (Article 3 of Te Tiriti; Reid et al., 2017; National Ethics Advisory Committee MoH, 2019; Ministry of Health NZ, 2019). The overall aim of our project was to improve Indigenous representation in cancer and }cardiovascular datasets generated from cutting-edge scientific techniques and technologies. We aimed to carry this study out in a collaborative manner that was culturally safe, respectful, and empowered those }participating in the study in their research contribution. We planned to recruit and }consent Māori participants coming in for appointments on suspicion of having breast or ovarian cancer, or coronary artery disease. For participants presenting with cancer, we planned to collect biopsy specimens }or tumor post-surgery and then generate 3D tumor organoids from these samples. Treatment of organoids with controls or relevant clinically used chemotherapies would then be carried out, and the response of these organoids to treatment would be investigated using scRNA-seq/snRNA-seq. We also planned to consent participants upon admission for coronary artery bypass surgery, to investigate the role of immune cells in pericardial and periaortic fat deposits in acute vs. stable coronary artery disease. While designing this project, it was clear that there were key aspects that could present as potential risks to Indigenous communities should we fail to integrate Indigenous worldviews and beliefs into all aspects of this work. These included: the project design, experimental procedures, analyses, and dissemination of data. To ensure that we were carrying out our study in a culturally safe manner, we further evaluated and engaged the wider community in the following specific areas: protection of samples going overseas, the use of live human tissue for organoid development, the use of RNA and transcriptional data and what that means for Indigenous participants, and the interpretation and governance of the data.

Here in this report, we present a real-world example of how consultation with community and the incorporation of Indigenous methodologies and worldviews, can be applied and implemented into molecular biology in a practical yet culturally responsive manner. This work builds upon previously published ethical frameworks (Beaton et al., 2017; Hudson et al., 2016a,b,c) and here we apply these to the newly emerging molecular technologies of tumor organoids and scRNA-seq/snRNA-seq (Table 1).

**Table 1:** Ethical frameworks based on tikanga Māori for live tissue and modern sequencing studies.

**Application of ethical frameworks for live tissue models, snRNAseq and associated Māori data**			
**Tika**	**Mana**	**Manaakitanga**	**Whakapapa**
**1. Initial planning and participant consent**			
Collaboration with experts in tikanga Māori, Māori researchers with expertise in cancer, cardiovascular disease, modern technologies	Participants consulted after appointment with medical professionals and as early as possible	Samples and data can be returned at the conclusion of the study	Recruitment of participants based on self-reported ethnicity
Solely Māori participant cohort (recruiting only those identifying as Māori)	Translations of titles in participant consent form with options for discussing the project in Te Reo Māori with members of the research team who are fluent	Participants approached as soon as possible (upon confirmation of biopsy or surgery) to allow time to discuss with whanau and community (or iwi)	Use of whanau or collective consent where the participant/whanau wishes (co-signature) → collective consent
Written consent needed for keeping samples beyond life of study or if other researchers want to access data or samples (participant to decide if they want data to be uploaded to databases etc.)	Māori governance group and supervisors as well as community groups active part of project planning, supporting and growing the Māori science workforce	Participant consent is preferably face to face (kanohi ki te kanohi) but if not possible email/phone	Continuous relationship building between researchers, participants, communities and clinicians
**2. Considerations for live tissue use and collection of tissue**			
Limitations to how the organoids are used for this study in place so participants only consenting to the study at hand	Tissue and organoids not kept beyond life of study	Tissue is tapu and a taonga, and has mauri associated with it → protected by kaitiaki, and recognized as koha	Organoids only kept animated for necessary duration to treat them and prepare for sequencing
Dewar blessed by kaumatua prior to storage of organoids and tissue	Tissue and organoids stored in dewars or freezers that only contain human tissue	Karakia said over samples written by kaumatua from Ngāti Toa Rangatira (or Christian/other prayer if preferred) → opt-out rather than opt-in	Tissue and corresponding waste streams kept separate, acknowledging the mauri and wairua associated with them
**3. Sending samples overseas to the Broad Institute of MIT and Harvard, Boston, USA**			
Organoids and live tissue not grown outside of Aotearoa	Samples that are not destroyed by analysis (SnRNAseq) are returned to Aotearoa at the conclusion of analyses	No samples or data stored at the Broad beyond the necessary time for analyses or beyond the time where the Māori researcher is present at the Broad	Samples taken to Boston alongside Māori researcher
Samples sent to the USA frozen or fixed for analysis at same time as Māori researcher leaving for USA	Tissue and organoids stored in freezers with only human tissue, sequenced on flow cells that only contain human tissue	Karakia before samples were sent for safe travel of samples; Karakia before analyses in the USA as well as Aotearoa (before sequencing or cell/nuclei isolation)	Māori researcher has kaitiakitanga (guardianship) over samples and data whilst at the Broad Institute
**4. Use of Māori data, data governance and dissemination**			
Data removed from Broad Institute servers and returned to Aotearoa and Aotearoa based storage services	Māori governance group and community members as authors on publications where appropriate	Participants have the option for secure return of individual data (including raw genetic data), individual lay summary results, or overall study results	Consistent engagement with iwi and community representatives
Māori governance group oversees and makes final decisions regarding use of data, and have veto rights over use of data.	Participants have sovereignty over their own data and tissue including snRNAseq data, use by the research team is only with their permission for publication of deidentified results	Ngāti Toa Rangatira community invited to share in findings at study conclusion, as well as clinical partners, and participant groups themselves (community groups, advocacy groups etc e.g., Hei $\bar{\text{A}}$huru Mowai)	Broad Institute research team have no rights to use the data without permission from the Aotearoa research team (memorandum of understanding in place)
Participants to choose whether to permit recontact about data sharing or further studies. Data is not shared with other researchers unless explicit consent is given to do so	Participants can choose to allow or not, for deposition of data into public databases		

Tika, mana, manaakitanga, and whakapapa are core terms as adopted from Te Ara Tika and the four pillars model described by Hudson et al. (2010).

## Consultation and collaboration processes to inform study design

Building relationships (whakawhanaunatanga) between researchers, communities, and participants, was critical for this research, particularly as it is grounded in working with Māori communities and participants. This reciprocity in the research process ensured both parties had mutual goals in research aims, and that both were satisfied with how the research would be conducted. Consultation in these settings should not be tokenistic, and therefore it is important that we fully developed collaborative relationships with other researchers working with Māori communities, other Māori academics, and Māori clinicians, as well as iwi and community groups (Table 2). It is important that this research was conducted in a “mana-enhancing” manner, a strength-based model that draws upon and enhances a person's presence or power (empowerment of the individual and others as opposed to power over an individual) and that of their community and family (Wilson et al., 2021). The mana of the participants contributing to this research as well as that of the Māori leaders assisting with this research should be acknowledged. Koha, a Māori custom where a gift/offering/contribution is given to acknowledge the contribution or service of an individual or individuals, was given to Māori advisors to acknowledge the time and knowledge they have gifted to this project.

**Table 2:** Relative roles of groups involved in this body of work.

**Group/relationships**	**Specific group role in the project**	**Form of engagement with group**
Māori governance group	Oversees project including dissemination, use of data, data interpretation, protection of data, final decisions on data usage	Quarterly meetings, meetings prior to dissemination of data, preferably kanohi ki te kanohi (face to face)
Supervisory group and Academic collaborators	Oversees scientific integrity of the project, ensures work is scientifically valid and performed to a high standard, provides supervision of the project	Regular meetings and sharing of data, direct contribution to producing data outputs
Broad Institute of MIT and Harvard collaborators	Providing expertise in modern sequencing technologies and translational research, supervision and fostering international collaboration, assistance with experimental and computational procedures	Monthly meetings and supervision where needed to assist with the project, visit to Aotearoa New Zealand at beginning of project's inception
Local Indigenous representatives (e.g., Ngāti Toa Rangatira)	Ensure steps taken in project are aligned with local Indigenous values and customs, and are best practice for the community and participants involved	Hui (meeting) at beginning of the project to gather feedback in response to the project, hui at end of project to discuss findings and next steps (or where is felt necessary by governance and academic teams)
Clinical partners at Te Whatu Ora Health New Zealand and Ora Toa	Ensuring scientific processes are aligned with participant healthcare, does not interfere with participant's healthcare, and provides clinical context for the project/ability to collect samples	Engagement at beginning of project to ensure project is feasible in the Wellington hospitals system; continuous engagement where appropriate throughout project as participant recruitment continues, dissemination of findings where appropriate
Patient advocacy groups/Partner Organizations	Provide patient voice in the project as well as expertise in health research needs (e.g., Hei $\bar{\text{A}}$huru Mowai provides Māori leadership, as does chief advisor Māori at Cancer Society/Heart foundation	Updated on results of the project prior to publishing to ensure experts in Māori health and science agree with what is to be published
Participants	Providing direct feedback on the project as active contributors, guide the use and distribution of data	Participants have sovereignty of the data and make ultimate decisions on what to use data for; participants are consulted at time of consent with options for recontact, dissemination of results, and further follow up (at their discretion)

Each group has a key important role in ensuring this research is done in a culturally safe manner that is best practice based on current frameworks and abilities.

Initial discussions surrounding the scope of the project were organized between the research group involved (both Indigenous and non-Indigenous representatives) and Māori experts in health leadership in the cancer and cardiovascular spaces. Initial advice was taken on from these discussions to frame the basis of ethics applications, including the study protocol and cultural practices (tikanga) developed within it, application for endorsement from a regional Māori advisory group (the Interim Research Advisory Group Māori, Te Whatu Ora Capital and Coast), and participant information sheets. Participant information sheets and consent forms were assessed by Indigenous researchers with expertise in Māori health research and sequencing based projects. This was to ensure consent forms implemented the tikanga Māori protocols designed for this project (where appropriate), were understandable to a lay audience, and prioritized the autonomy of the individual or whānau (family) consenting to the research. As a result of the consultation process, several key changes were made to our protocols. Firstly, we used an opt-out process rather than an opt-in process for karakia (traditional Māori chant or prayer) that was said over the samples at the beginning and end of each day when samples were used. The option for Christian or traditional karakia was given to participants, as well as a karakia written by Ngāti Toa Rangatira kaumatua (elders) blessing the sample and the researcher. Secondly, we ensured that tissue was kept both separate from non-human tissue (e.g., in −80°C freezers that contained only human samples) as well as separated between different participants, with the inclusion of waste streams (i.e., not culturing cells from different individuals together and using separate containers for cell culture waste streams). These measures were used to acknowledge the body and all associated parts of the body being tapu, or sacred, and having whakapapa (genealogy) and mauri (spiritual lifeforce) associated with them (Hudson et al., 2016b,c, 2010). Thirdly, ensuring comparisons were not made to non-Indigenous individuals by only recruiting Indigenous participants to the study. The aim of this study was to increase Indigenous representation in modern biomedical datasets rather than to directly compare Indigenous to non-Indigenous people. It was also considered important to reduce potential for deficit framing [“identification of internal deficiencies as the cause of disparities, focusing on Indigenous culture or peoples as the problem” (Reid and Robson, 2007)] to prevent conclusions from being drawn that could contribute to and potentially exacerbate Indigenous health inequities (Wright et al., 2022). Previous work by Indigenous researchers demonstrate that deficit narratives focused on the “problems that need to be fixed” for Indigenous individuals, and foster victim blaming and cultural deficit explanations rather than focusing on the root causes of disparities like colonialism and racism (Curtis et al., 2023; Bullen et al., 2023; Mashford-Pringle and Pavagadhi, 2020). By increasing Indigenous representation in these datasets and models, we are generating information that can be used to help improve Indigenous health outcomes without reference to non-Indigenous people as the standard.

Further consultation with clinicians involved in this work was undertaken to ensure the project was feasible within the New Zealand healthcare system and processes and would not affect the participant's healthcare. Clinical teams were made aware of the study as early as possible, and were made aware of how the samples were to be used (i.e., for the use of live tissue models). Samples were only to be collected if the clinician involved (radiologist, surgeon, or pathologist) deemed that collection would not interfere with diagnostic processes (i.e., tissue for diagnosis was prioritized for cancer specimens), and would not cause the participant any harm (i.e., if the participant had increased risk of bleeding with biopsy tumor samples were not taken, and samples from participants undergoing coronary artery bypass surgery was only what was removed as part of the medical procedure). This ensured that patient welfare was not compromised by the research.

This project, whilst based in Aotearoa New Zealand, has been designed in collaboration with researchers based at the Broad Institute of Massachusetts Institute of Technology (MIT) and Harvard University (Boston, USA). The Broad Institute has world-class experts in generating and analyzing scRNA-seq and snRNA-seq datasets. Sending samples from Māori participants overseas to be analyzed in collaboration with experts, under the protection of Indigenous researchers, ensured that the benefits of these techniques could be maximized. It was therefore important that the USA-based research team had a comprehensive understanding of why the study was designed as it was and the concerns of the Māori participants donating their samples and data to the study. A hui (meeting) was organized based at the University of Otago Wellington site, with researchers from the Broad Institute traveling from the USA to Aotearoa (New Zealand) to partake and share (wānanga) in different aspects of the project. In Te Ao Māori (the Māori world), important meetings are always conducted kanohi ki te kanohi (using a face-to-face approach); in this way, researchers are seen, known, and acknowledged within the community, allowing for stronger reciprocal relationships to be formed and building trust between the researchers and the participants contributing to the research (Hudson et al., 2007). This hui involved a 2-day symposium where researchers from across Aotearoa, with expertise in cardiovascular disease, breast cancer, ovarian cancer, and Māori health and tikanga, shared their expertise. Discussion on the ethics surrounding translational research, and the tikanga protocols in relation to this project allowed formulation of a better practice model for working in a mana-enhancing manner with Māori patient communities. For example, the use of traditional prayer, or karakia, for wet lab-based work, discussions on the role of family and community in the consent process, the use of live tissue and its implications for Indigenous peoples, the risks associated with this type of sequencing and the possibility of doing this work in an overseas laboratory, were focal to the discussions had. It is important to note that whilst a best practice model for working with our participant cohort was strived for, this is a continuous process as understandings deepen and practicalities shift. This discussion informed changes in the study design and identified challenges that could not be fully solved in this study alone, such as how to best approach multiplexing of participant samples during the sequencing process, or what Indigenized governance of the data would look like in best practice. These challenges would be continuously worked at following these initial discussions and continuously throughout this study. For example, the physical storage and translocation of sequencing data was discussed but was further adapted later once the research was being conducted on site at the Broad institute, ensuring that mechanisms for data transfer and storage were practical and able to be conducted by the Māori research team involved in a feasible manner. Other things such as the use of whole genome sequencing was discussed but was decided that it would not be performed as this required further ethical considerations and was outside of the scope of this study. Consideration of these comments allowed adaptations of all ethics forms and patient consent sheets accordingly.

In addition to the hui above, the manuhiri (visitors) from the Broad Institute and the Aotearoa based research team visited Takapuwāhia marae (a meeting complex of the iwi or local tribe) to discuss the project with mana whenua (a tribe with authority over the unceded land and territory), Ngāti Toa Rangatira. The visitors experienced a traditional Māori welcome onto the marae known as a powhiri, for discussions surrounding the project and the methods and methodology used for it. Kaumātua (respected elders), those involved in leadership groups in the health and education space, and other interested community members were involved. This hui allowed relationships to be built between the research team and the iwi, as well as both institutions (University of Otago, Wellington and Te Rūnanga o Toa Rangatira). Kai (food) was shared, and time for whakawhanaunatanga (Māori concept involving building relationships through shared experiences and working together, establishing a sense of belonging and relational connections) between the leaders but also the rangatahi (youth) from the iwi was prioritized. Koha in the form of a monetary gift was given to acknowledge the time and space given by the iwi for guests to come on to their marae and whenua (land). Conversations surrounding how we could continue to build this relationship and kaupapa were had. For example, discussions on how the research group could support future Ngāti Toa Rangatira medical students and scientists, and on health research priorities for iwi members.

Discussions surrounding the research project were also had with local general practitioners (primary care physician) at Ora Toa, the health providers run through Te Rūnanga o Toa Rangatira. Reciprocal support in research endeavors between University of Otago Wellington and Ora Toa centers was essential in building relationships and ensuring the wellbeing of participants was priority, as some of the participants in this study would be coming through Ora Toa centers on their healthcare journeys. General practitioners became aware of the research through these discussions and could notify potential participants about the research as early as possible, allowing another potential avenue for participants to have maximal time to talk with whānau and others about the project.

This project was intentionally designed to be led by a Māori researcher (the first author) whom is also a part of the Ngāti Toa Rangatira community. This allowed for greater levels of trust between the research team and the local iwi involved in the project as all discussions were built on these relationships. The research team also aimed to mitigate any potential conflicts by establishing governance groups early on, by ensuring reciprocal relationships were built, and acknowledging the time and involvement of the community through use of koha. Members of the community who were actively involved have been given authorship to acknowledge the wealth of traditional knowledge that has been shared with the research group for this project.

## Cultural considerations for recruitment, the use of live tissue models and RNA-sequencing

*Tika*, meaning “correct” or “right” is a term from a Māori worldview that regards the study design and its goals. To ensure that the research encompasses tika we implemented into the study design that experts in the appropriate fields for this study, including Indigenous health, Māori customs, cancer and cardiovascular disease and others, were a continuous part of the collaboration process throughout our study. This includes a Māori governance group, as well as Māori academics and researchers in both traditional and non-traditional settings. The Māori governance group will oversee and make final decisions regarding the participant data that is used in this study and any interpretations made of data; they have veto rights over any dissemination or use of data that they deem not appropriate. Local Indigenous representatives, such as members of local iwi/tribal groups in Wellington New Zealand, Ngāti Toa Rangatira, will also be consulted. Previous meetings with these groups/individuals have covered topics such as plans for the dissemination of data and what should be shared with the wider research community and plans for taking samples overseas. These groups will also contribute to discussions surrounding publication of data prior to doing so ensuring that any conclusions made are appropriate and the interests of participants and the wider Māori community are protected (Table 2). Further to this, written consent will be needed should the samples be kept beyond the life of this study, or should other researchers want to access the data or samples. Importantly the current study is a solely Indigenous cohort, allowing us to focus entirely on a Māori voice informing research that involves organoid development and scRNA-seq and snRNA-seq. This ensures that benefits of such technologies can be maximized for Māori (Reid et al., 2017). Te Tiriti o Waitangi guarantees that the crown, and therefore its entities, will act in a way that Māori are not disadvantaged and if they are, will take measures to correct this imbalance (Reid et al., 2017). Whilst Māori make up 16% of the Aotearoa population, the dominant voice in research is that of non-Māori, and thus by solely recruiting a Māori cohort we hope to share the Māori voice and way of being through this project.

Although there is no direct translation in English, *Mana* is loosely defined as the “presence” of a person, place or object, the spiritual power or the authority and status. In this context, we associated Mana in relation to research that is just and equitable, and “mana enhancing” for the individuals contributing to our research. For example, ensuring that participants have fully informed consent is an important aspect of this, and the outcome of research needs to be shared with all who contribute, unless they decide otherwise. Consistent and prolonged engagement throughout the study and beyond with mana whenua is crucial to ensuring research goals of the scientific team and iwi are reciprocal, open, honest, equitable and most importantly, meaningful. We aim to uphold the mana of participants by making sure they are as involved in decisions as possible, consenting in the clinic as early as possible alongside family/whānau for the discussion if they wish, and ensuring they feel appropriately informed, that the decision is theirs to make, and that they have time to discuss with others if they desire. In this study participants were approached as early as possible (upon confirmation the participant would be having a procedure) before their surgical or biopsy procedure. This was ideally in-person when attending an outpatient appointment, or over the phone/email in the case that no other form of initial contact was possible. For consent, participants were consulted after their scheduled appointments with their medical team, with the research team liaising with the participant's clinical care team to ensure the patient would be comfortable and in a good state of mind for participation in research, ensuring patient welfare was at the forefront of the research team's priorities. We aim to give participants as much time as possible, while acknowledging that we are also working within the constraints of how the Westernized hospital system works and how appointment lists are generated. Consequently, there are occasions where recruitment to the study does not allow for as much time for the participant as we would like. However, informed discussions with general practitioners and community allowed us to make this kaupapa as safe as possible for participants to consent on a shorter timeline; we also aim to follow up with participants who said they are happy to be recontacted to give them the opportunity to withdraw from the study should they decide to, and to have any data and tissue returned. This approach to consent allows participants to learn about the study, take the time where possible to discuss with whānau, friends and iwi members, before deciding whether to participate, and to consider the decision to participate later. The intent is to treat consent as a living agreement with participants where they have rangatiratanga over use of their tissue and data. Finally, Māori researchers with experience conducting research alongside Māori communities, advised translations of titles in te reo Māori (the Māori language) in the patient consent forms was appropriate rather than the entire consent document. Translation of titles was outsourced to professionals fluent in the language. There was an option for oral translation and korero (discussion) in Te Reo Māori with members of the research team who are fluent if the participant wished.

*Whakapapa* is paramount to many parts of Māori culture and biological research, and can be linked to both relationships and genealogy, describing the relationship with one another but also relationships to one's ancestors, to the land and environment around us, and to other living things. Importantly, in the Māori world view there is no concept of the colonial construct of blood quantum, instead knowledge of one's whakapapa is the only prerequisite to self-identify as Māori; if you have whakapapa Māori, you are Māori. It is culturally inappropriate to impose concepts such as blood quantum onto individuals participating in research (Cram, 2019; Cram et al., 2022), and so in this study potential participants were identified based on self-reported ethnicity in their clinical records and upon discussion with the participant. This self-identification also allows for self-determination (rangatiratanga) for those who whakapapa Māori or have Indigenous ethnicity. Relationships between researchers, participants, communities and clinicians can all also be considered whakapapa, as whakapapa is about how people relate to each other and work together, extending beyond blood connections to the building of relationships. Whakapapa is also important in one's overall holistic health, and therefore it is important to acknowledge whānau or family in the decision-making processes for participants in research (Durie, 1982). During consultation participants were also informed that they could nominate another person, such as a member of their whānau, to make decisions on their behalf regarding the use of data generated from this work, emphasizing the mana family holds and the importance of whakapapa in making decisions in the consent process. Whakapapa also goes beyond oneself and whānau with the notion of collective consent, recognizing the authority and role of wider familial or community groups in Indigenous cultures, particularly in issues surrounding consent. Collective good traditionally is prioritized over individual autonomy in Māori culture, and in a research setting, is complimentary to individual consent, particularly in situations where genetic information is being gathered. For some individuals, this collective decision-making is therefore an extremely important part of the consenting process. This study provides an option for whānau/family consent, particularly as live tissue and genetic materials (RNA) are being used. Participants have the option to have co-signatures for consenting to the project, as well as the option for a co-signatory to act as a point of contact should the participant themselves be unable to be contacted by the research team. Ethical frameworks are still largely Westernized and often exist in the scope of academic settings. Legally, consent is still an individual's right to choose, and thus the option for whānau consent has been used as a way of acknowledging Māori beliefs within the constraints of the Westernized and academic systems.

*Manaakitanga* in the research context describes the responsibility of research to be culturally and socially safe and sensitive (Hudson et al., 2010). This includes ensuring use of Māori protocols and tikanga were appropriate and treating these concepts with respect. For this research project, tumor organoids are generated from breast or ovarian cancer samples, removed at the time of surgery by a member of the patient's clinical team, at the pathologist/radiologist's discretion. In Māori culture, tissue (anything derived from a person including blood, fluids, tissue) is tapu, or comes with restrictions, and is recognized as a taonga, a treasure. This extends to any nucleic acid and data related to that tissue. As there is tapu associated with the tissue, appropriate processes for donating tissues for research ought to be considered. Hudson and colleagues have previously described the traditional concept of tākoha as an appropriate process for the gifting of consented taonga and the responsibility of its care, doing so with respect and transparency. Tākoha obligates whoever uses the taonga, in this case the tissue, and all things derived from the tissue including sequencing and clinical data, to deliver useful outcomes in a culturally safe manner (Caron et al., 2020). This is explained further in frameworks such as Te Mara Ira (Hudson et al., 2016a), where in genomic research tākoha and Te Hau o te Taonga (the spirit of the gift), reflect the responsibility to make decisions regarding the taonga that adheres to the informed consent that the participant gave, respects the spirit it was given in, and relates to the level of information that the participant was given throughout the consent process. The research team in this context has a duty of care to the participant donating tissue and associated data to this project, to their whānau (family), and to the samples, throughout the lifetime of this project. As an example, the mauri or life force of a resource, in this case the patient tissue samples, should be maintained and protected by kaitiaki (guardians). Further, pericardial fat is being used to investigate the immune cell landscape in coronary artery disease. Whilst these tissues will not be cultured, they too are recognized as tapu and a taonga, particularly being heart tissue, and need to be treated with respect and as a treasure. In this way, participation toward this research is essentially the ultimate koha, or gift, and should be treated as such by researchers. For this work, we sought guidance from kaumatua and leaders from Ngāti Toa Rangatira, other Māori researchers and clinicians, and from whānau and iwi for the development of tikanga for the laboratory. This included the use of karakia in the laboratory, the physical separation of participant samples for use in the laboratory as acknowledgment of the mauri and wairua they have, the use and importance of whānau consent processes, and data sovereignty guidelines. To ensure kaitiakitanga of the samples whilst traveling to the USA, the samples were accompanied by the Māori researcher leading this project who had the responsibility of ensuring their safe departure and arrival. A karakia written by elders from the local iwi/tribe for this study to say over samples, acknowledges the sample as a gift and the spiritual lifeforce that comes with it. This karakia was used throughout the project at the beginning and end of working with the samples (each day the samples were used), and in the case where participants preferred, other karakia or Christian prayer was also used. A karakia was used before samples traveled to the USA to bless them for safe travel.

Organoids can be grown and passaged similarly to cell line models and can be kept animated for extended periods of time. To acknowledge the wairua (spirit) that these models have, and that they too have whakapapa and are tapu the organoids developed in this study will only be kept animated for the necessary duration to treat them and prepare for sequencing (<6 months). Limitations to how they will be used for this study have been put in place to ensure that participants are consenting only to the study at hand, as to not exploit the tissue and related data stemming from its use, further protecting from any commercial interests. This has been outlined in the study protocol and patient consent forms that are received by ethics committees and the participants themselves, respectively.

It was also important to the research group that when using Māori samples, we needed to maximize the benefit for our participants and whānau that were contributing to the research. To achieve equity in health research, researchers need to be creative regarding ways translational research is conducted with the resources available in Aotearoa, the resources available globally, and the responsibility to Te Tiriti and to mana whenua that the research is being conducted responsibly. This project could not have been achieved without the support from researchers at the Broad Institute of MIT and Harvard in Boston, USA, who have given resources, time and knowledge to this work to ensure maximum benefits can be achieved from using single cell/nuclei RNA sequencing on these samples, in a safe and responsive manner. The Broad institute team are experts in single nuclei RNA sequencing, both with wet-lab experimental procedures, particularly with traditionally difficult to work with samples (small biopsy samples, fat samples, etc.), and the corresponding computational analyses of these datasets. Currently, this level of expertise in this area is unavailable in Aotearoa. Without this international collaboration, we would not have been able to use this kind of technology for our Māori participants. Developing these international relationships is key to how we can continue to uplift our Māori participants and uphold our responsibility to Te Tiriti, but only if the relationships are based on trust and understanding. Whilst we acknowledge the risk of sending samples overseas, when the communities, participants, the research team and the Māori research lead involved are aware of the risks and are actively mitigating them (by ensuring cultural protocols are embedded throughout), communities will benefit from translational research conducted through international relationships. Further, the team at the Broad Institute were open and receptive to using the tikanga protocols developed for the work in Aotearoa at the Broad Institute also, and have adapted storage space, and experimental and computational procedures to ensure practices at the Broad align with the practices performed in Aotearoa for this project. This includes allowing access to the sequencing platform for karakia before sequencing begins, ensuring samples are stored with human tissue only, and ensuring the transfer of data to Aotearoa based servers for data storage. Whilst the initial plan was to transport the samples to the Broad on-person, restrictions due to weight limitations and dry ice shipping meant that samples had to be sent ahead of the Māori researcher. Samples were blessed prior to their departure and sent at approximately the same time as the Māori researcher leaving for the USA. Therefore, whilst we aimed to have samples under the protection of the Māori researcher during transfer to the Broad, we had to be realistic in terms of both physical and legal restrictions of sending biological samples overseas over long distances. It is important to note that traditional tikanga Māori protocols develop in “new ways that keep pace with world changes,” as written by Māori academic Professor Hirini Mead; tikanga Māori has the capacity to make pragmatic changes and apply traditional practice to modern situations as the research environment changes and as technology progresses (Mead, 2016).

This work aims to emphasize the role of collaboration in these research environments, highlighting the importance of the Broad Institute researchers visiting Aotearoa for early project meetings and to experience Māori culture and understand why concepts such as tikanga are so important in translational research. With the Broad team adapting some of their laboratory protocols to ensure this project could be done according to the tikanga aforementioned, we hope to have highlighted that international collaborations with Indigenous samples can be done if, and only if, trusting relationships are developed between both research groups, and particularly the communities involved.

## Indigenous data sovereignty

The technology of scRNA-seq and snRNA-seq enables each cell or nucleus to be “captured” in a small volume where they are lysed, and the contents of each individual cell sequenced (Brazovskaja et al., 2019). There is the potential for interpretation or possible extrapolation of whakapapa information (ancestry information) from this sequence data, and thus the challenge remains in how this data can and should be interpreted and what sort of protections should surround the data to mitigate these risks. To address this to the best of current capabilities and ensure our research is tika and performed with manaakitanga and mana, a data management plan was developed with Indigenous sovereignty and protection of this data at the forefront. This is an ever-evolving area of research and thus in this study, Indigenous data sovereignty has been ensured to the best of the research team's abilities at the time of publishing, while acknowledging that as progress is made, changes in how to address the use of Māori data should also change.

Māori data sovereignty refers to the governance, quality, and use of Māori data (Hudson et al., 2020; Jansen, 2016; Kukutai and Cormack, 2020; Lovett et al., 2019). Māori data should be subject to Māori governance, and should have tribal sovereignty, realizing the aspirations of iwi and hapu (Jansen, 2016; Kukutai and Cormack, 2020; Lovett et al., 2019; Hudson et al., 2017). Data for and about Māori should be safeguarded and protected to prevent misuse or harm and should be of high quality and integrity. Further to this, per Article 2 of Te Tiriti o Waitangi, Māori have tino rangatiratanga (self-determination, sovereignty) over their taonga which in this case is interpreted as both the tissue samples themselves and the data generated from these tissues, and therefore it is our responsibility as Māori and non-Māori to ensure we are upholding Te Tiriti o Waitangi through our scientific and data analysis practices. In this study, participants were consented for the generation of scRNA-seq or snRNA-seq data of patient-derived tumor organoid samples, either untreated or treated with gold-standard/potential therapies, or from adipose tissues surrounding the heart. Previous cases of misuse of Indigenous genetic data have resulted in false claims being made, and have provided opportunities for therapeutic development without the insights gained from research with Indigenous populations benefiting Indigenous communities (Fox, 2020). For example, in the 1980's the Havasupai Tribal Nation in Arizona, USA, experienced misuse of their genetic data where data that was originally sought to address high levels of diabetes in their communities, was misused for unrelated studies on schizophrenia, migration, and consanguinity, subjects that were considered “taboo” for the tribe (Garrison et al., 2019). In Aotearoa, the warrior gene is the most notorious example of misuse of genetic research, where use of the term “warrior” was used to hypothesize connections between migration routes, aggressive behavior, and health outcomes related to smoking cessation for Māori populations, despite the genetic variant found being present in all populations, not just Māori (Garrison et al., 2019). Unsurprisingly, this association of a specific phenotype with Māori created the false narrative of Māori being genetically aggressive, and a huge amount of distrust in subsequent genetic research for Māori communities. Further to this, it is standard scientific practice internationally to deposit deidentified sequencing data into public databases for access by other researchers at the time of publication. However, this practice is not consistent with Indigenous data sovereignty models (Tsosie et al., 2021a). To address these concerns, we have designed a model where participants in this study have ownership/self-determination over their own data and tissue, including scRNA-seq and snRNA-seq data, and use by the research team is only with their permission for scientific publication of deidentified results. This model has been adapted from prior Māori-led genomic research, using a model that not only includes participant-retained ownership and determination of data with permission for the research team to use the data for only the purposes outlined in the study, but also community informed and governance group directed use of data to ensure that all data-related decisions are made with accuracy and cultural safety in mind. This practically means that participants can choose to allow for deposition of data into public databases, or not, at the time of consenting. Options for declining data deposition outright, or approval to be re-contacted about data sharing later for specific projects or purposes, has been included into the consent process. Data therefore will not be shared with other researchers unless explicit permission is received from the participants, with any additional use needing additional consent and ethical approval. All other details of the participants would not be shared without patient consent. Community and governance group decisions would include things such as ensuring scientific conclusions from the research are sound and accurate.

For the work performed in the USA at the Broad Institute of Harvard and MIT, a memorandum of understanding agreement with the Broad-based research team has been put in place to confirm that data would not be stored long term at the Broad Institute and all data generated from scRNA-seq and snRNA-seq experiments would be returned to Aotearoa. The US-research team have no rights to use the data without permission from the NZ research team.

All decisions for use of the data generated from this body of work would first be in collaboration with the Māori governance group for this project, a team of experts in tikanga Māori, health, and science. This is to ensure use of the data was culturally safe, and that all conclusions made from the work are beneficial for participants and Māori communities, reducing risk of any harms and misdemeanor. Whilst the wider use of data would be decided at the level of the individual (i.e., whether data could be collected and what type of data, whether sequencing data would be used for database depository or not etc.), the use of generalized deidentified data for purposes such as dissemination of data to the scientific community or to Māori communities would be at the discretion of the governance group based on their expertise in these areas to provide extra protection of the data. Further consultation and collaboration where appropriate with local iwi or participants (anonymously where possible) will be done when needed to confirm participants are comfortable with all conclusions from the work. Participants also have the option for individual data including raw transcriptional data (on a password protected hard drive or appropriate secure cloud-based transfer) to be returned to them when generated, as well as the option for any tissue remaining at the end of the study to be returned to them. For participants that wish to have individual lay summary results returned to them, copies of the publications the data was used for alongside a lay summary will be sent by email or mail. This could be in conjunction with an individual phone call or visit from the researchers to ensure the results have been understood and allow opportunity for questions. We also previously acknowledged that the samples used in this study and the associated data from these samples are taonga (gifts/treasures) that the research team have been given permission to use for this study. Returning of the gift, te whakahoki i te taonga, is an important step in this process to acknowledge both the gift of the tissue and the data from the participants and the gift of the knowledge that has been shared with the research team by community members and leaders. An example of how we aim to return the gift in this sense is through hui to share findings and continue to build relationships with iwi and community, and authorship of community members on publications in acknowledgment of the knowledge shared for this work. In addition to regular meetings with the Māori governance group for this work, we have also planned for further dissemination of findings to community groups, clinical partners/treating clinicians, and the Ngāti Toa Rangatira community prior to any publication. This is to ensure experts in Māori health and science, as well as the communities involved, are comfortable with what is being published. Leaders such as the Māori cancer leadership group Hei $\bar{\text{A}}$huru Mowai, the cancer society of New Zealand, and the chief advisor Māori of the New Zealand Heart foundation will also be updated on results from this project. Overall, these practices were put in place to ensure Māori were fairly represented, and their mana upheld.

## Conclusions

Eliminating health inequities globally looks like meaningful engagement in Indigenous health initiatives, shared leadership, mutual understanding of attitudes and barriers toward equity, and creating a culture of collaboration. We present here a study that carries out genuine community engagement and implements Indigenous protocols to inform and underpin a scientific study design that prioritizes the cultural safety of Māori participants, enhances the mana of those involved, and creates biomedical research models that are consistent with Indigenous worldviews.

**Figure 1:**
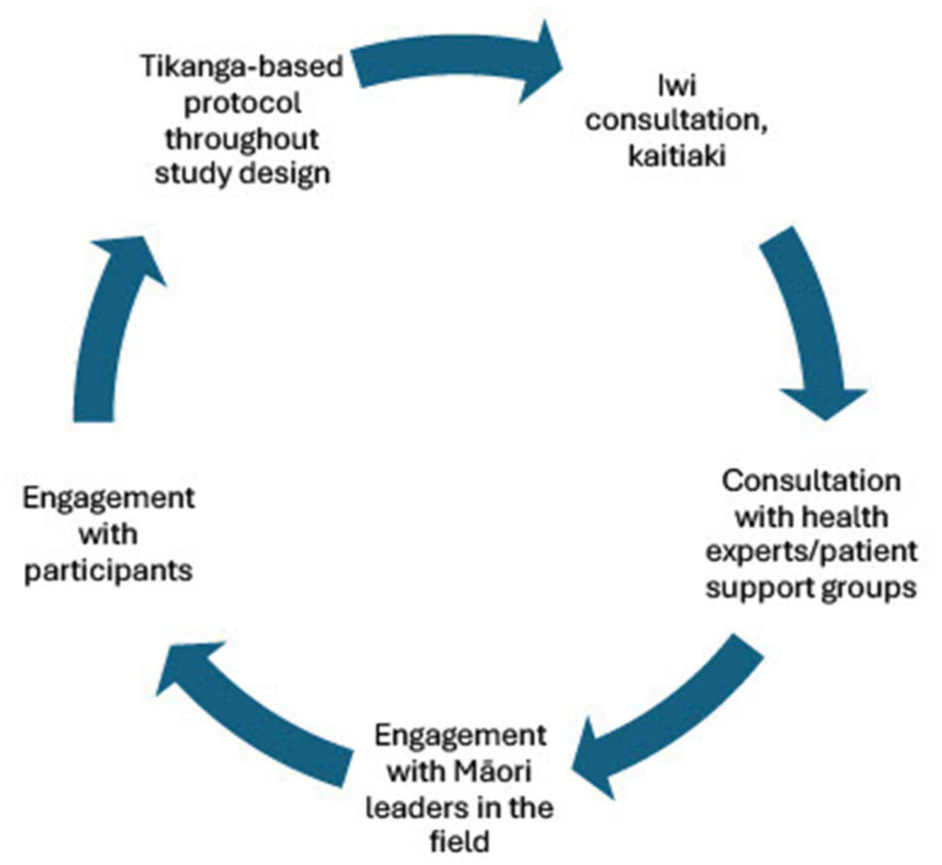
Cycle of engagement for studies working with Māori participants to ensure meaningful translational research is conducted that benefits the communities we are trying to serve. Engagement with Māori academics, leaders, communities, iwi, and participants is essential to ensure the Māori customs and practices that are put in place for both wet-lab and computational procedures and best practice.

We propose a model (Figure 1) of continuous collaborative consultation with Indigenous communities, health experts and patient support groups, Indigenous experts in the appropriate fields, and participants themselves (Table 2), to inform appropriate protocols that are respectful to the individuals and whānau (families) who are contributing to research. Although this work has been conducted in the context of breast and ovarian cancer, and coronary artery disease, we aim to provide a practical application of using existing ethical frameworks for modern biomedical research with Indigenous communities, to highlight how practices can be adapted to better suit the patient cohorts contributing to the research (Table 1). It is important to acknowledge that the processes outlined are what was deemed important for this study based on the collaboration and engagement between Indigenous communities in Wellington, New Zealand, and the research team. It is encouraged that each study should do their own consultation and form collaborative relationships to perform culturally appropriate research with their own local Indigenous communities. Different Indigenous groups will share different views on some of the issues outlined in this study. This body of work should not be used as a definitive method of incorporating these values into scientific protocols but as an example of some of the ways they can be incorporated into science practically, based on engagement and relationships formed with communities and Indigenous researchers. Further, this is a continuous cycle of engagement and Indigenization of traditional western science research, and therefore continued engagement with local representatives and experts and development of protocols with Māori customs embedded will be done to ensure that we continue to conduct research that is culturally safe and best practice for the participants involved (Figure 1). Establishment of these relationships allows opportunities for further Indigenous scholars to access high quality institutes in a culturally safe way, to ensure progress continues to be made toward equitable precision research. As the current study progresses, we will continue to adapt and improve these practices as ethical frameworks progress and develop, ensuring these cultural processes develop in line with the scientific protocols used.

Research with Indigenous communities is crucial to ensure existing entrenched inequities are eliminated from biomedical research, however any research that is performed alongside Indigenous communities should be research that the community themselves needs and wants and upholds their tino rangatiratanga in all aspects. Overall, we have designed a study that is built upon Indigenous values, in the Aotearoa New Zealand context. We have implemented traditional Māori customs into our research to the best of current capabilities with the aim of continuing to improve Indigenous representation in biomedical research and create better practice models for clinical research.

## Data Availability

The original contributions presented in the study are included in the article/supplementary material, further inquiries can be directed to the corresponding author.

## References

[B1] Amrute J. M. Lai L. Ma P. Koenig A. L. Kamimoto K. Bredemeyer A.. (2023). Defining cardiac functional recovery in end-stage heart failure at single-cell resolution. Nat. Cardiovasc. Res. 2, 399–416. 10.1038/s44161-023-00260-8 37583573 PMC10426763

[B2] Aragam K. G. Jiang T. Goel A. Kanoni S. Wolford B. N. Atri D. S.. (2022). Discovery and systematic characterization of risk variants and genes for coronary artery disease in over a million participants. Nat. Genet. 54, 1803–1815. 10.1038/s41588-022-01233-6 36474045 PMC9729111

[B3] Aramoana J. Koea J. Comm N. C. (2020). An integrative review of the barriers to indigenous peoples participation in biobanking and genomic research. JCO Glob. Oncol. 6, 83–91. 10.1200/JGO.18.00156 32213083 PMC7853871

[B4] Beaton A. Hudson M. Milne M. Port R. V. Russell K. Smith B.. (2017). Engaging Maori in biobanking and genomic research: a model for biobanks to guide culturally informed governance, operational, and community engagement activities. Genet. Med. 19, 345–351. 10.1038/gim.2016.111 27632687

[B5] Beyene K. Chan A. H. Y. Naslund P. Harrison J. (2021). Patient-related factors associated with oral anticoagulation control: a population-based cohort study. Int. J. Pharm. Pract. 29, 443–450. 10.1093/ijpp/riab041 34302345

[B6] Bi J. Newtson A. M. Zhang Y. Devor E. J. Samuelson M. I. Thiel K. W.. (2021). Successful patient-derived organoid culture of gynecologic cancers for disease modeling and drug sensitivity testing. Cancers 13:2901. 10.3390/cancers13122901 34200645 PMC8229222

[B7] Boretto M. Maenhoudt N. Luo X. Hennes A. Boeckx B. Bui B.. (2019). Patient-derived organoids from endometrial disease capture clinical heterogeneity and are amenable to drug screening. Nat. Cell Biol. 21, 1041–1051. 10.1038/s41556-019-0360-z 31371824

[B8] Brazovskaja A. Treutlein B. Camp J. G. (2019). High-throughput single-cell transcriptomics on organoids. Curr. Opin. Biotechnol. 55, 167–171. 10.1016/j.copbio.2018.11.002 30504008

[B9] Bullen J. Hill-Wall T. Anderson K. Brown A. Bracknell C. Newnham E. A.. (2023). From deficit to strength-based aboriginal health research-moving toward flourishing. Int. J. Environ. Res. Publ. Health 20:75395. 10.3390/ijerph20075395 37048008 PMC10094537

[B10] Caron N. R. Chongo M. Hudson M. Arbour L. Wasserman W. W. Robertson S.. (2020). Indigenous genomic databases: pragmatic considerations and cultural contexts. Front. Publ. Health 8:111. 10.3389/fpubh.2020.00111 32391301 PMC7193324

[B11] Chaffin M. Papangeli I. Simonson B. Akkad A. D. Hill M. C. Arduini A.. (2022). Single-nucleus profiling of human dilated and hypertrophic cardiomyopathy. Nature 608, 174–180. 10.1038/s41586-022-04817-8 35732739 PMC12591363

[B12] Claw K. G. Anderson M. Z. Begay R. L. Tsosie K. S. Fox K. Garrison N. A.. (2018). A framework for enhancing ethical genomic research with Indigenous communities. Nat. Commun. 9:2957. 10.1038/s41467-018-05188-3 30054469 PMC6063854

[B13] Claw K. G. Dorr C. R. Woodahl E. L. (2024). Implementing community-engaged pharmacogenomics in Indigenous communities. Nat. Commun. 15:920. 10.1038/s41467-024-45032-5 38296967 PMC10831049

[B14] Connor H. D. (2019). Whakapapa back: mixed indigenous Māori and Pākehā genealogy and heritage in Aotearoa/New Zealand. Genealogy 3:73. 10.3390/genealogy3040073

[B15] Cram F. (2019). “Kaupapa Māori health research,” in Handbook of Research Methods in Health Social Sciences, ed. P. Liamputtong (Singapore: Springer Singapore), 1507–1524.

[B16] Cram F. Adcock A. Liamputtong P. (2022). Handbook of Qualitative Cross-Cultural Research Methods: A Social Science Perspective. Chapter 4: Kaupapa Māori Research (Cheltenham: Edward Elgar Publishing), 56–84.

[B17] Curtis E. Jones R. Tipene-Leach D. Walker C. Loring B. Paine S.. (2019). Why cultural safety rather than cultural competency is required to achieve health equity: a literature review and recommended definition. Int. J. Eq. Health 18:174. 10.1186/s12939-019-1082-3 31727076 PMC6857221

[B18] Curtis E. Jones R. Willing E. Anderson A. Paine S. J. Herbert S.. (2023). Indigenous adaptation of a model for understanding the determinants of ethnic health inequities. Discov. Soc. Sci. Health 3:10. 10.1007/s44155-023-00040-6

[B19] Curtis E. Loring B. Harris R. McLeod M. Mills C. Scott N.. (2022). Inform Development of The Interim New Zealand Health Plan (iNZHP).

[B20] de Witte C. J. Espejo Valle-Inclan J. Hami N. Lõhmussaar K. Kopper O. Vreuls C. P. H.. (2020). Patient-derived ovarian cancer organoids mimic clinical response and exhibit heterogeneous inter- and intrapatient drug responses. Cell Rep. 31:107762. 10.1016/j.celrep.2020.107762 32553164

[B21] Ding S. Chen X. Shen K. (2020). Single-cell RNA sequencing in breast cancer: understanding tumor heterogeneity and paving roads to individualized therapy. Cancer Commun. 40, 329–344. 10.1002/cac2.12078 32654419 PMC7427308

[B22] Durie M. (1994). Whaiora, Maori Health Development. Auckland: Oxford University Press, 67–81.

[B23] Fan Y. Zhou H. Liu X. Li J. Xu K. Fu X.. (2021). Applications of single-cell RNA sequencing in cardiovascular research. Front. Cell Dev. Biol. 9:810232. 10.3389/fcell.2021.810232 35174168 PMC8841340

[B24] Fox K. (2020). The illusion of inclusion—the “all of us” research program and indigenous peoples' DNA. N. Engl. J. Med. 383, 411–413. 10.1056/NEJMp1915987 32726527

[B25] Garnier S. Harakalova M. Weiss S. Mokry M. Regitz-Zagrosek V. Hengstenberg C.. (2021). Genome-wide association analysis in dilated cardiomyopathy reveals two new players in systolic heart failure on chromosomes 3p25.1 and 22q11.23. Eur. Heart J. 42, 2000–2011. 10.1093/eurheartj/ehab030 33677556 PMC8139853

[B26] Garrison N. A. Hudson M. Ballantyne L. L. Garba I. Martinez A. Taualii M.. (2019). Genomic research through an indigenous lens: understanding the expectations. Annu. Rev. Genom. Hum. Genet. 20, 495–517. 10.1146/annurev-genom-083118-015434 30892943

[B27] Garvey G. Cunningham J. (2019). “Social inequalities and cancer in Indigenous populations,” in Reducing Social Inequalities in Cancer: Evidence and Priorities for Research, eds. S. Vaccarella, J. Lortet-Tieulent, R. Saracci, D. I. Conway, K. Straif, and C. P. Wild (Lyon: IARC Scientific Publications), 79–86.

[B28] Griffiths K. Coleman C. Lee V. Madden R. (2016). How colonisation determines social justice and Indigenous health-a review of the literature. J. Popul. Res. 33, 9–30. 10.1007/s12546-016-9164-1

[B29] Gwynne K. Jiang S. Venema R. Christie V. Boughtwood T. Ritha M.. (2023). Genomics and inclusion of Indigenous peoples in high income countries. Hum. Genet. 142, 1407–1416. 10.1007/s00439-023-02587-5 37479894 PMC10449672

[B30] Henare K. L. Parker K. E. Wihongi H. Blenkiron C. Jansen R. Reid P.. (2019). Mapping a route to Indigenous engagement in cancer genomic research. Lancet Oncol. 20, e327–e335. 10.1016/S1470-2045(19)30307-9 31162106

[B31] Henderson L. M. Robinson R. F. Ray L. Khan B. A. Li T. Dillard D. A.. (2019). VKORC1 and novel CYP2C9 variation predict warfarin response in Alaska Native and American Indian People. Clin. Transl. Sci. 12, 312–320. 10.1111/cts.12611 30821933 PMC6510382

[B32] Hudson M. Anderson T. Dewes T. K. Temara P. Whaanga H. Roa T.. (2017). ““He Matapihi ki te Mana Raraunga”-Conceptualising Big Data Through a Māori Lens,” in He Whare Hangarau Maori—Language, Culture & Technology, eds. H. Whaanga, T. T. A. G. Keegan, and M. Apperley (Hamilton: Te Pua Wananga ki te Ao/Faculty of Maori and Indigenous Studies, The University of Waikato), 64–73.

[B33] Hudson M. Beaton A. Milne M. Port W. Russell K. Smith B.. (2016a). Te Mata Ira: Guidelines for Genomic Research with Māori. Te Mata Hautu Taketake—Maori & Indigenous Governance Centre, University of Waikato, Hamilton, New Zealand.

[B34] Hudson M. Beaton A. Milne M. Port W. Russell K. Smith B.. (2016c). He Tangata Kei Tua: Guidelines for biobanking with Māori. Waikato: Māori and Indigenous Governance Centre, University of Waikato.

[B35] Hudson M. Garrison N. A. Sterling R. Caron N. R. Fox K. Yracheta J.. (2020). Rights, interests and expectations: Indigenous perspectives on unrestricted access to genomic data. Nat. Rev. Genet. 21, 377–384. 10.1038/s41576-020-0228-x 32251390

[B36] Hudson M. Milne M. Reynolds P. Russell K. Smith B. (2010). Te ara tika: Guidelines for Maori Research Ethics: a Framework for Researchers and Ethics Committee Members. Auckland: Health Research Council of New Zealand on behalf of the Putaiora Writing Group.

[B37] Hudson M. Southey K. Uerata L. Beaton A. Milne M. Russell K.. (2016b). Key informant views on biobanking and genomic research with Māori. New Zealand Med. J. 129, 29–42. 27977650

[B38] Hudson M. L. Ahuriri-Driscoll A. L. M. Lea M. G. Lea R. A. (2007). Whakapapa—a foundation for genetic research? J. Bioethical Inq. 4, 43–49. 10.1007/s11673-007-9033-x

[B39] Jansen R. (2016). Indigenous data sovereignty: a Māori health perspective. Indigenous Data Sovereignty 193:11. 10.22459/CAEPR38.11.2016.11

[B40] Kalayinia S. Goodarzynejad H. Maleki M. Mahdieh N. (2018). Next generation sequencing applications for cardiovascular disease. Ann. Med. 50, 91–109. 10.1080/07853890.2017.1392595 29027470

[B41] Kim J. Koo B.-.K Knoblich J. A. (2020). Human organoids: model systems for human biology and medicine. Nat. Rev. Mol. Cell Biol. 21, 571–584. 10.1038/s41580-020-0259-3 32636524 PMC7339799

[B42] Kukutai T. Cormack D. (2020). “*Pushing the Space”: Data Sovereignty and Self-determination in Aotearoa NZ. Indigenous Data Sovereignty and Policy* (London: Routledge), 21–35.

[B43] Litvinukova M. Talavera-Lopez C. Maatz H. Reichart D. Worth C. L. Lindberg E. L.. (2020). Cells of the adult human heart. Nature 588, 466–472. 10.1038/s41586-020-2797-4 32971526 PMC7681775

[B44] Liu J. Ma P. Lai L. Villanueva A. Koenig A. Bean G. R.. (2022). Transcriptional and immune landscape of cardiac sarcoidosis. Circ. Res. 131, 654–669. 10.1161/CIRCRESAHA.121.320449 36111531 PMC9514756

[B45] Lovett R. Lee V. Kukutai T. Cormack D. Rainie S. C. Walker J.. (2019). Good data practices for Indigenous data sovereignty and governance. Good Data. 2019, 26–36.

[B46] Martin A. R. Kanai M. Kamatani Y. Okada Y. Neale B. M. Daly M. J.. (2019). Clinical use of current polygenic risk scores may exacerbate health disparities. Nat. Genet. 51, 584–591. 10.1038/s41588-019-0379-x 30926966 PMC6563838

[B47] Mashford-Pringle A. Pavagadhi K. (2020). Using OCAP and IQ as frameworks to address a history of trauma in indigenous health research. Am. Med. Assoc. J. Ethics 22, E868–E873. 10.1001/amajethics.2020.868 33103649

[B48] Matheson K. Bombay A. Anisman H. (2018). Culture as an ingredient of personalized medicine. J. Psychiatry Neurosci. 43, 3–6. 10.1503/jpn.170234 29252161 PMC5747533

[B49] Mead H. M. (2016). Tikanga Maori (Revised Edition): Living by Maori Values. Wellington: Huia Publishers.

[B50] Mills M. C. Rahal C. A. (2019). scientometric review of genome-wide association studies. Commun. Biol. 2:9. 10.1038/s42003-018-0261-x 30623105 PMC6323052

[B51] Ministry of Health NZ (2019). Wai 2575 Māori Health Trends Report.

[B52] Mudd-Martin G. Cirino A. L. Barcelona V. Fox K. Hudson M. Sun Y. V.. (2021). Considerations for cardiovascular genetic and genomic research with marginalized racial and ethnic groups and Indigenous peoples: a scientific statement from the American Heart Association. Circ. Genom. Precis. Med. 14:e000084. 10.1161/HCG.0000000000000084 34304578

[B53] Nagaraj S. H. Toombs M. (2021). The gene-drug duality: exploring the pharmacogenomics of Indigenous populations. Front. Genet. 12:687116. 10.3389/fgene.2021.687116 34616423 PMC8488351

[B54] Narasimhan V. Wright J. A. Churchill M. Wang T. Rosati R. Lannagan T. R. M.. (2020). Medium-throughput drug screening of patient-derived organoids from colorectal peritoneal metastases to direct personalized therapy. Clin. Cancer Res. 20:73. 10.1158/1078-0432.CCR-20-0073 32376656 PMC8366292

[B55] National Ethics Advisory Committee MoH New Zealand. (2019). National Ethical Standards for Health and Disability Research and Quality Improvement. Wellington: Ministry of Health.

[B56] Need A. C. Goldstein D. B. (2009). Next generation disparities in human genomics: concerns and remedies. Trends Genet. 25, 489–494. 10.1016/j.tig.2009.09.012 19836853

[B57] Nguyen M. T. Gallagher C. Pitman B. M. Emami M. Kadhim K. Hendriks J. M.. (2020). Quality of warfarin anticoagulation in Indigenous and non-indigenous australians with atrial fibrillation. Heart Lung Circul. 29, 1122–1128. 10.1016/j.hlc.2019.11.006 31980393

[B58] Ortega V. E. Meyers D. A. (2014). Pharmacogenetics: implications of race and ethnicity on defining genetic profiles for personalized medicine. J. Allergy Clin. Immunol. 133, 16–26. 10.1016/j.jaci.2013.10.040 24369795 PMC3933289

[B59] Parikh V. N. Ashley E. A. (2017). Next-generation sequencing in cardiovascular disease: present clinical applications and the horizon of precision medicine. Circulation 135, 406–409. 10.1161/CIRCULATIONAHA.116.024258 28137961 PMC5310819

[B60] Patrinos G. P. Quinones L. A. Sukasem C. (2023). Editorial: pharmacogenomics and ethnicity: prevalence and clinical significance of pharmacogenomic biomarkers in indigenous and other populations. Front. Pharmacol. 14:1180487. 10.3389/fphar.2023.1180487 37063283 PMC10090656

[B61] Popejoy A. B. Fullerton S. M. (2016). Genomics is failing on diversity. Nature 538, 161–164. 10.1038/538161a 27734877 PMC5089703

[B62] Porter R. J. Murray G. I. McLean M. H. (2020). Current concepts in tumour-derived organoids. Br. J. Cancer 123, 1209–1218. 10.1038/s41416-020-0993-5 32728094 PMC7555542

[B63] Reichart D. Lindberg E. L. Maatz H. Miranda A. M. A. Viveiros A. Shvetsov N.. (2022). Pathogenic variants damage cell composition and single cell transcription in cardiomyopathies. Science 377:eabo1984. 10.1093/eurheartj/ehac544.2992 35926050 PMC9528698

[B64] Reid P. Paine S. J. Curtis E. Jones R. Anderson A. Willing E.. (2017). Achieving health equity in Aotearoa: strengthening responsiveness to Maori in health research. N. Z. Med. J. 130, 96–103. 29121628

[B65] Reid P. Robson B. (2007). Understanding Health Inequities (Hauora: Maori Standards of Health IV A Study of the Years 2000–2005), 4.

[B66] Reweti A. Ware F. Moriarty H. (2023). A tangata whenua (people of the land) approach to conceptualising Māori health and wellbeing. Glob. Health Promot. 30, 11–18. 10.1177/17579759221130948 36314287

[B67] Roberts M. (2013). Ways of seeing whakapapa. Sites J. Soc. Anthropol. Cult. Stud. 10, 93–120. 10.11157/sites-vol10iss1id236

[B68] Roselli C. Chaffin M. D. Weng L. C. Aeschbacher S. Ahlberg G. Albert C. M.. (2018). Multi-ethnic genome-wide association study for atrial fibrillation. Nat. Genet. 50, 1225–1233. 10.1038/s41588-018-0133-9 29892015 PMC6136836

[B69] Saeki S. Kumegawa K. Takahashi Y. Yang L. Osako T. Yasen M.. (2023). Transcriptomic intratumor heterogeneity of breast cancer patient-derived organoids may reflect the unique biological features of the tumor of origin. Breast Cancer Res. 25:21. 10.1186/s13058-023-01617-4 36810117 PMC9942352

[B70] Samarasinghe S. R. Hoy W. Jadhao S. McMorran B. J. Guchelaar H. J. Nagaraj S. H.. (2023). The pharmacogenomic landscape of an Indigenous Australian population. Front. Pharmacol. 14:1180640. 10.3389/fphar.2023.1180640 37284308 PMC10241071

[B71] Segelov E. Garvey G. (2020). Cancer and Indigenous populations: time to end the disparity. JCO Glob. Oncol. 6, 80–82. 10.1200/JGO.19.00379 32031442 PMC6998017

[B72] Selak V. Rahiri J. L. Jackson R. Harwood M. (2020). Acknowledging and acting on racism in the health sector in Aotearoa New Zealand. N. Z. Med. J. 133, 7–13. 32994633

[B73] Simonson B. Chaffin M. Hill M. C. Atwa O. Guedira Y. Bhasin H.. (2023). Single-nucleus RNA sequencing in ischemic cardiomyopathy reveals common transcriptional profile underlying end-stage heart failure. Cell Rep. 42:112086. 10.1016/j.celrep.2023.112086 36790929 PMC10423750

[B74] Sisman Y. Schnack T. Høgdall E. Høgdall C. (2022). Organoids and epithelial ovarian cancer—a future tool for personalized treatment decisions? Mol. Clin. Oncol. 16:29. 10.3892/mco.2021.2462 34987799 PMC8719262

[B75] Spagnol G. Sensi F. De Tommasi O. Marchetti M. Bonaldo G. Xhindoli L.. (2023). Patient derived organoids (PDOs), Extracellular matrix (ECM), Tumor microenvironment (TME) and drug screening: state of the art and clinical implications of ovarian cancer organoids in the era of precision medicine. Cancers 15:2059. 10.3390/cancers15072059 37046719 PMC10093183

[B76] Stevens A. A. (2008). Different way of knowing: tools and strategies for managing Indigenous knowledge. De Gruyter. Libri. 58, 25–33. 10.1515/libr.2008.003

[B77] Tawfik S. M. Elhosseiny A. A. Galal A. A. William M. B. Qansuwa E. Elbaz R. M.. (2023). Health inequity in genomic personalized medicine in underrepresented populations: a look at the current evidence. Funct. Integr. Genom. 23:54. 10.1007/s10142-023-00979-4 36719510

[B78] Te Aho o Te Kahu C. C. A. (2023). Rongohia Te Reo, Whatua He Oranga: The Voices of whānau Māori Affected By Cancer. Wellington: Cancer Control Agency.

[B79] Te Rito J. S. (2007). Whakapapa: a framework for understanding identity. MAI Rev. LW 1:10. Available at: https://www.journal.mai.ac.nz/system/files/maireview/56-65-1-PB.pdf

[B80] Tremblay M. C. Olivier-D'Avignon G. Garceau L. Échaquan S. Fletcher C. Leclerc A. M.. (2023). Cultural safety involves new professional roles: a rapid review of interventions in Australia, the United States, Canada and New Zealand. Int. J. Indigenous Peopl. 19, 166–175. 10.1177/11771801221146787

[B81] Tsosie K. S. Fox K. Yracheta J. M. (2021a). Genomics data: the broken promise is to Indigenous people. Nature 591:529. 10.1038/d41586-021-00758-w 33742179

[B82] Tsosie K. S. Yracheta J. M. Kolopenuk J. A. Geary J. (2021b). We have “gifted” enough: Indigenous genomic data sovereignty in precision medicine. Am. J. Bioeth. 21, 72–75. 10.1080/15265161.2021.1891347 33825628

[B83] Tucker N. R. Chaffin M. Fleming S. J. Hall A. W. Parsons V. A. Bedi K. C. Jr.. (2020). Transcriptional and cellular diversity of the human heart. Circulation 142, 466–482. 10.1161/CIRCULATIONAHA.119.045401 32403949 PMC7666104

[B84] Tuveson D. Clevers H. (2019). Cancer modeling meets human organoid technology. Science 364:952. 10.1126/science.aaw6985 31171691

[B85] Wilson D. Moloney E. Parr J. M. Aspinall C. Slark J. (2021). Creating an Indigenous Māori-centred model of relational health: a literature review of Māori models of health. J. Clin. Nurs. 30, 3539–3555. 10.1111/jocn.15859 34046956 PMC8597078

[B86] Wright K. Dehar A. Stott N. S. Mackey A. Sorhage A. Tapera R.. (2022). Prioritizing indigenous health equity in health registers: an environmental scan of strategies for equitable ascertainment and quality data. Glob. Health Res. Pol. 7:24. 10.1186/s41256-022-00250-6 35854338 PMC9295285

